# Where next for telehealth? Reflections from the 2nd International Congress on Telehealth and Telecare

**DOI:** 10.5334/ijic.923

**Published:** 2012-06-15

**Authors:** Nick Goodwin

**Affiliations:** Senior Fellow, The King’s Fund, London, UK E-mail: ngoodwin@kingsfund.org.uk

**Keywords:** e-health, evidence, innovation, review, telehealth, telecare

On the 6–8 March 2012, The King’s Fund and the University Medical Centre Utrecht hosted the second International Congress on Telehealth and Telecare that successfully brought together policy-makers, academics, professionals, users, innovators and industry from countries from all over the world both face-to-face and online. During the three-day event, some 500 delegates, speakers and sponsors were in attendance with another 600 online delegates. The twitter reach of the congress (#kft12) was also significant appearing on 131,000 people’s timelines via 1500 tweets!

This conference supplement provides the abstracts of the many scientific papers—plenary, sessional and poster—that were delivered. What did we learn over the three days?

## Day 1—The importance of high quality research evidence

The first day of the congress focused on problems with designing and implementing studies that evaluate complex interventions, such as telehealth. We learned that past evidence has been characterised by varying standards in the quality of research, and that the wide range of methods and measures used has not enabled a comprehensive assessment of its impact to be made.

In a prescient debate, our discussions focused on the range of dilemmas that are faced when trying to interpret research findings from past evidence. For example, from my own overview of the collective impact of telehealth that was presented [1] we showed that the majority (about two-thirds) of studies have had a positive impact across a range of impact criteria (including cost-effectiveness). However, studies varied in the type of technology used (simple and cheap, to complex and expensive); the diseases being treated (single or multiple, physical and mental); the client being supported (e.g., age, wealth, ethnicity); and the context of where it was carried out (e.g., rural or urban). The sheer heterogeneity of the evidence makes comparison and generalization problematic.

As a result of these difficulties there is a lack of understanding of the relative impact of telehealth from other models of long-term care. More importantly, we are bereft of good evidence for how to successfully apply and adopt new technologies in practice. The first day concluded, therefore, that better and more consistent approaches to evaluation were needed.

## Day 2—Emerging laws for successful telehealth adoption

The second day of the congress started with direct messages of the UK Government’s support for telehealth from keynote presentation from Rt. Hon. Paul Burstow, MP—the Health Minister—and from Stephen Johnson, Head of Long Term Conditions at the Department of health. Drawing on the headline findings from the UK’s Whole System Demonstrators trial—the largest cluster randomised control trial (RCT) of telehealth ever conceived involving 6000 patients across three different localities [2]—it was argued that not only it was ‘morally right’ to offer such care to people who would benefit but that it was also the smart thing to do in reducing the cost-burden on the care system in the long-term.

This vision was subsequently supported by the three sites of the evaluation—Cornwall, Kent and Newham—who were all keen to roll-out telehealth based on the lessons they had gained during the pilot process. The apparent coming together of belief and science was music to the ears of the converted.

As the day progressed many stories of the issues, problems and successes with using new technologies were told. These stories began to draw out a set of key ‘laws’ for successful telehealth adoption internationally of which my top five (in no particular order) were as follows:

1. **Keep it simple** for patients and carers to use and for professionals to adopt
2. **Tailor the service** to the specific needs of the user, including how they might best use and accept new technology
3. **Enhance human contact** by better connecting patients to family, friends and care professionals so they and their carers feel safe, secure and empowered
4. **Embed an IT infrastructure** to act as the bedrock of better care through integrated information systems
5. **Build relationships and networks** to influence behaviours, build alliances, and overcome the significant mismatch of motives that exist between patients, carers, professionals, commissioners and industry.

The last of these is arguably the most important task. There is an acute need in all care systems to work across a diversity of competing interests towards more mutually beneficial relationships that meet the common cause of improving people’s lives.

## Day 3—An undermining of belief, the speed of new innovation

Day 3 will be remembered for the carefully crafted and well delivered presentation on the cost-effectivenes of telehealth in the Whole System Demonstrator the trial by Catherine Henderson from the LSE. As a colleague commented, watching it live on video, the world of telehealth was seemingly turned upside down within 5 crucial minutes that demonstrated how the undoubted benefits to patients from the trial came at a significant price (more than £80,000 per QALY). Much debate ensued, with a key focus on whether and how the high direct costs identified in the trial (e.g., the cost of the kit and the processes involved in its deployment) could be reduced through new and better approaches. Knee-jerk reaction to the findings must be withheld, however, until the full peer-reviewed studies are published in the BMJ.

I will also remember the day for the wide range of new innovations in telehealth that were presented—particular those using smart phones, tablet computers, and the internet—which make those technologies deployed in the trail look old hat. Also, new interfaces connecting patients, carers and families directly with their medical records and advice from nurses and doctors is just around the corner. This will turn traditional ways of working on their head—a case of Dr Finlay’s Facebook rather than Casebook (thank you Stephen from Philips for that one!). I will remember two papers in particular—the ‘vision’ for how to achieve this in Singapore (from Tikki Gee, MOH Holdings, Singapore), and the poster-winning presentation from Israel on the ‘reality’ of implementing such an approach from Israel (from Dr Orit Jacobsen, Clalit Health Services).

## Conclusions

Whilst the capabilities of new technologies to solve our problems in this field move apace, the research evidence lags behind this by a number of years. But it is the glacial (if not geological) progress of innovation in health and social care systems that is most problematic. As Prof. Guus Schrijvers argued on Day 1, what is needed is ‘simultaneous innovation’ when the redesign of our care system to meet the challenges of an ageing society with complex needs can develop at a pace where new innovations can become an integral part of the process. Unlikely, of course, but the problems shines a light on the fact that 90% of the problem here is cultural, behavioural and systemic.

Learning the right lessons from the evidence is important and a deep dive into the findings is required since, as the keynote speech by Adam Darkins showed from his work in the VA system in the US, huge benefits at both a patient and system level are possible. The key lessons, as Adam described, are to build alliances and networks and also focus on the high volume and low cost solutions. Not attempting to ‘build a pyramid from the top-down’ (i.e., by focusing solely on the complex, high-cost, patients where interventions are expensive and difficult to deploy) is a key message that must be learned.
